# Tracking a Medically Important Spider: Climate Change, Ecological Niche Modeling, and the Brown Recluse (*Loxosceles reclusa*)

**DOI:** 10.1371/journal.pone.0017731

**Published:** 2011-03-25

**Authors:** Erin E. Saupe, Monica Papes, Paul A. Selden, Richard S. Vetter

**Affiliations:** 1 Paleontological Institute and Department of Geology, University of Kansas, Lawrence, Kansas, United States of America; 2 Center for Limnology, University of Wisconsin Madison, Madison, Wisconsin, United States of America; 3 Natural History Museum, London, United Kingdom; 4 Department of Entomology, University of California Riverside, Riverside, California, United States of America; Field Museum of Natural History, United States of America

## Abstract

Most spiders use venom to paralyze their prey and are commonly feared for their potential to cause injury to humans. In North America, one species in particular, *Loxosceles reclusa* (brown recluse spider, Sicariidae), causes the majority of necrotic wounds induced by the Araneae. However, its distributional limitations are poorly understood and, as a result, medical professionals routinely misdiagnose brown recluse bites outside endemic areas, confusing putative spider bites for other serious conditions. To address the issue of brown recluse distribution, we employ ecological niche modeling to investigate the present and future distributional potential of this species. We delineate range boundaries and demonstrate that under future climate change scenarios, the spider's distribution may expand northward, invading previously unaffected regions of the USA. At present, the spider's range is centered in the USA, from Kansas east to Kentucky and from southern Iowa south to Louisiana. Newly influenced areas may include parts of Nebraska, Minnesota, Wisconsin, Michigan, South Dakota, Ohio, and Pennsylvania. These results illustrate a potential negative consequence of climate change on humans and will aid medical professionals in proper bite identification/treatment, potentially reducing bite misdiagnoses.

## Introduction

**Figure 1:**
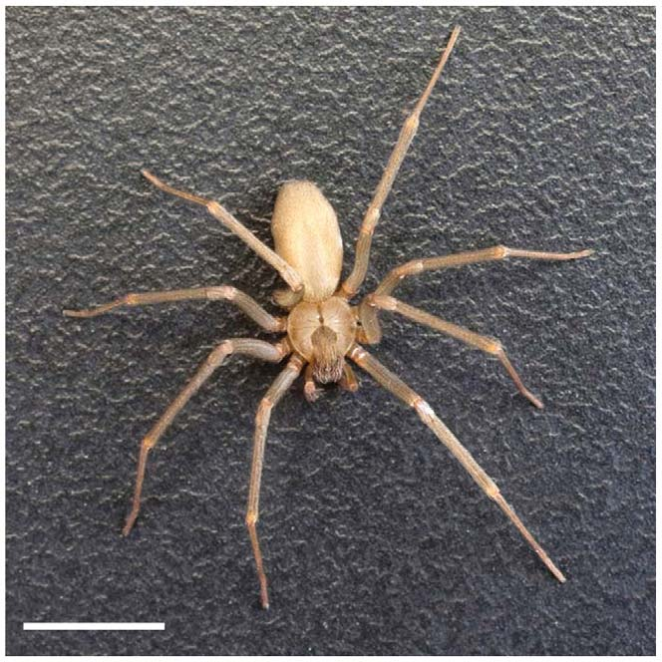
Brown recluse specimen. Collected in Lawrence, KS, USA. Scale: 5 mm.

The brown recluse spider (*Loxosceles reclusa*) (Fig. 1) is notorious for its necrosis-inducing bite [1]. Its venom contains a rare toxin, sphingomyelinase D, which, when incorporated into the skin and subcutaneous tissues, ultimately triggers platelet aggregation, endothelial hyperpermeability, hemolysis, and neutrophil-dependent skin necrosis [2]–[3].

**Figure 2:**
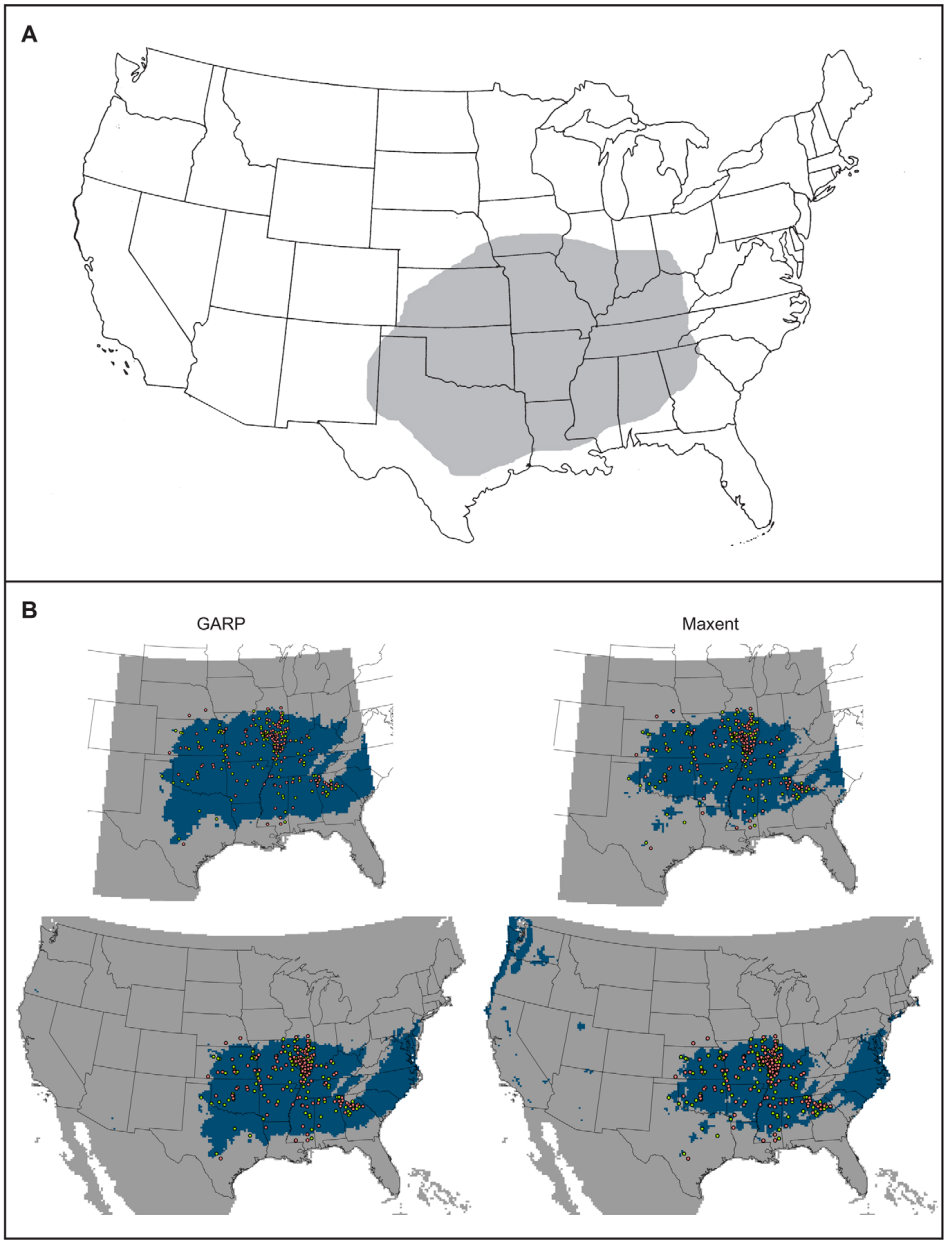
Present-day niche modeling results in comparison to previously identified distribution. A) Distribution of the brown recluse based on field studies and literature surveys from [4]. Note the general congruence between the niche model results and the distribution recognized by arachnologists. B) GARP models are depicted on the left, with the training region above and projection below. Maxent models are depicted on the right, with the training region above and projection below. Occurrence points are mapped onto these models, with lime green points = training data and salmon points = testing data. A threshold has been applied, allowing for a maximum of five percent omission error based on presence data. Results are depicted in USA Contiguous Albers Equal Area Conic map projection.

The species is synanthropic over much of its range in the United States and, as such, is commonly misconstrued as being ubiquitous throughout the country, particularly by medical professionals [4]–[5]. This leads to bite misdiagnoses for potentially serious conditions, such as Lyme disease, lymphoma, squamous cell carcinoma, and fungal infections [6]. Although habitation with humans may impact the range of *L. reclusa*, clear distributional demarcation does exist [4], [7]–[9]. The species is primarily found in the south-central United States, from southern Illinois south to Texas and from eastern Tennessee west to Kansas (Fig. 2A). *Loxosceles reclusa* prefers dry, dark areas, and outside of human habitation, is often found under stones and within the bark of dead trees [10].

In this study we applied the technique of ecological niche modeling (ENM) to distributional data, recorded over more than 10 years, with the aims of: (i) establishing the geographic range of the species with greater accuracy, and (ii) forming predictions of how the distribution might change as a result of climate warming trends.

ENM is a rich area of study that has seen tremendous growth in past years [11]–[14]. Species geographic occurrence points and predictor variables (usually climatic or environmental parameters) are related within machine-learning algorithms to make inferences about the environmental requirements for a species, which can then be projected onto geography. These models can also be projected onto changed landscapes, such as future climate scenarios, as a forecasting tool for biotic responses to differing conditions [15]–[18].

A growing number of studies indicate ENM is a useful tool for understanding ecological and geographic dimensions of vectors and disease reservoirs [19]–[27] and for identifying areas that may become affected under future climates, causing public health problems [28]–[32]. The more accurate range estimations and predictions for the brown recluse presented in this study could prove to be valuable to the medical community when assessing putative spider bites. Moreover, assessment of the future range of *L. reclusa* will prepare the public for potential invasions and alert them to the appropriate protocols for dealing with this spider.

## Results and Discussion

Ecological niches were generated using two programs: the Genetic Algorithm for Rule-set Prediction (GARP) [33] and Maxent [34] (see Methods section *b* for details). Seven environmental variables and 240 occurrence points served as the input data for model analysis (Methods sections *c* & *d*).

### (a) Accuracy metrics

Geographic projections of the ecological niche models were tested using the partial Receiver Operating Characteristic analysis (ROC) [35]–[36]. ROC is a threshold-independent measure of model quality as compared to null expectations. Partial ROC area under the curve (AUC) ratios were 1.49 and 1.45 for Maxent and GARP, respectively. The ratios were statistically significant above the line of null expectations (z-tests, P<0.001). The false negatives rate [35] was also calculated, with only 16 test points (11%) omitted from the Maxent model and 10 points (6.7%) omitted from the GARP model. Results from both analyses suggest high model fidelity and predictability.

Model results were also compared to previously published distributions of the brown recluse spider [4] (Fig. 2A). Experts have a fairly accurate knowledge of the current range of the species because of its medical importance, but in a qualitative sense, not quantitative as presented here. It is important to note that ENMs are often difficult to test and validate in this way (i.e., by comparison to the realized distribution of the species), because the actual distribution may not mirror the potential distribution (the niche model). Historical and biological barriers may prevent a species from occupying all suitable habitat [13], [37].

### (b) Present-day Modeling

Results from our present-day study mirror the known distribution of the brown recluse fairly well (Fig. 2). Discrepancies between the models and the previously recorded range include the suitable habitat predicted present in the Atlantic coast states (from New Jersey to South Carolina), whereas the easternmost extent of the present range of the brown recluse is currently documented to be Kentucky, Tennessee, and the south-western part of Ohio [4]. This incongruence either indicates model failure, with models including regions not ecologically suitable for the species, or the models are correct, and the brown recluse is not found on the east coast because of historical or biological barriers or limited dispersion potential, the latter scenario making range expansions plausible if these limitations are overcome [13], [38].

General congruence between the geographical range documented by arachnologists and our niche models (Fig. 2) suggests the partial synanthropy of the brown recluse is not the dominant influence on distributional patterns. Although the spider may be able to expand beyond its natural constraints with the aid of human infrastructure, the species is not widespread, as would be so if its only confines were buildings. For example, *L. reclusa* is not found north of a demarcation line cutting the state of Illinois approximately in half [39], yet houses are obviously present above this line (i.e., within the largest city in Illinois, Chicago). In this case, what is likely responsible for this demarcation line is the cold temperature tolerance of the brown recluse [39]. The scope of this study was to generate models that retrieved ecological signal for the spider, and even if the brown recluse does cohabit with humans, the issue is not overly concerning for our analysis.

In general, Maxent models predicted a smaller suitable area and had a less uniform coverage compared to the models produced with GARP. For example, the present-day Maxent projection estimated suitable ecological conditions in 33 states (counting the District of Columbia) with 13.68 percent of area within the continental U.S. predicted habitable. The present-day GARP projection only predicted suitable habitat in 25 states but predicted 15.89 percent of area within the continental U.S. as habitable (Fig. 2B). This may be a reflection of the underlying mechanics of Maxent, where the algorithm can give very large probability distribution values for environmental conditions outside the range present in the study area (called clamping) [34]. Clamping occurs when a pixel possesses a value for a variable outside of the range of values encountered in the training region. That pixel is given the closest value present for that variable in the model; however, the model is prone to over-extensive predictions when the response curve is high (or was increasing) and curtailed by the environment present in the training region. In our study, a few small areas of California, Oregon and Washington were specified as suitable in the Maxent models but were also designated as clamped regions.

There is considerable public ignorance about the brown recluse spider and its range within the United States. In a nation-wide survey [40], 1,406 out of 1,773 (79%) specimens submitted as *L. reclusa* did not belong to that species; all but two of the genuine brown recluse occurrences came from within the known area of distribution of the species. Of those two odd records, one (a singleton) could be explained by recent transport with household effects, while the other (3 specimens) lies within the Atlantic coast region, which may represent suitable recluse habitat. The results of our present-day study provide further confirmation that *L. reclusa* has a well-demarcated distribution, outside which it is unlikely to occur under normal circumstances. Thus, diagnoses and reports of brown recluse bites in non-endemic areas should be treated with skepticism.

### (c) Future Modeling

**Figure 3:**
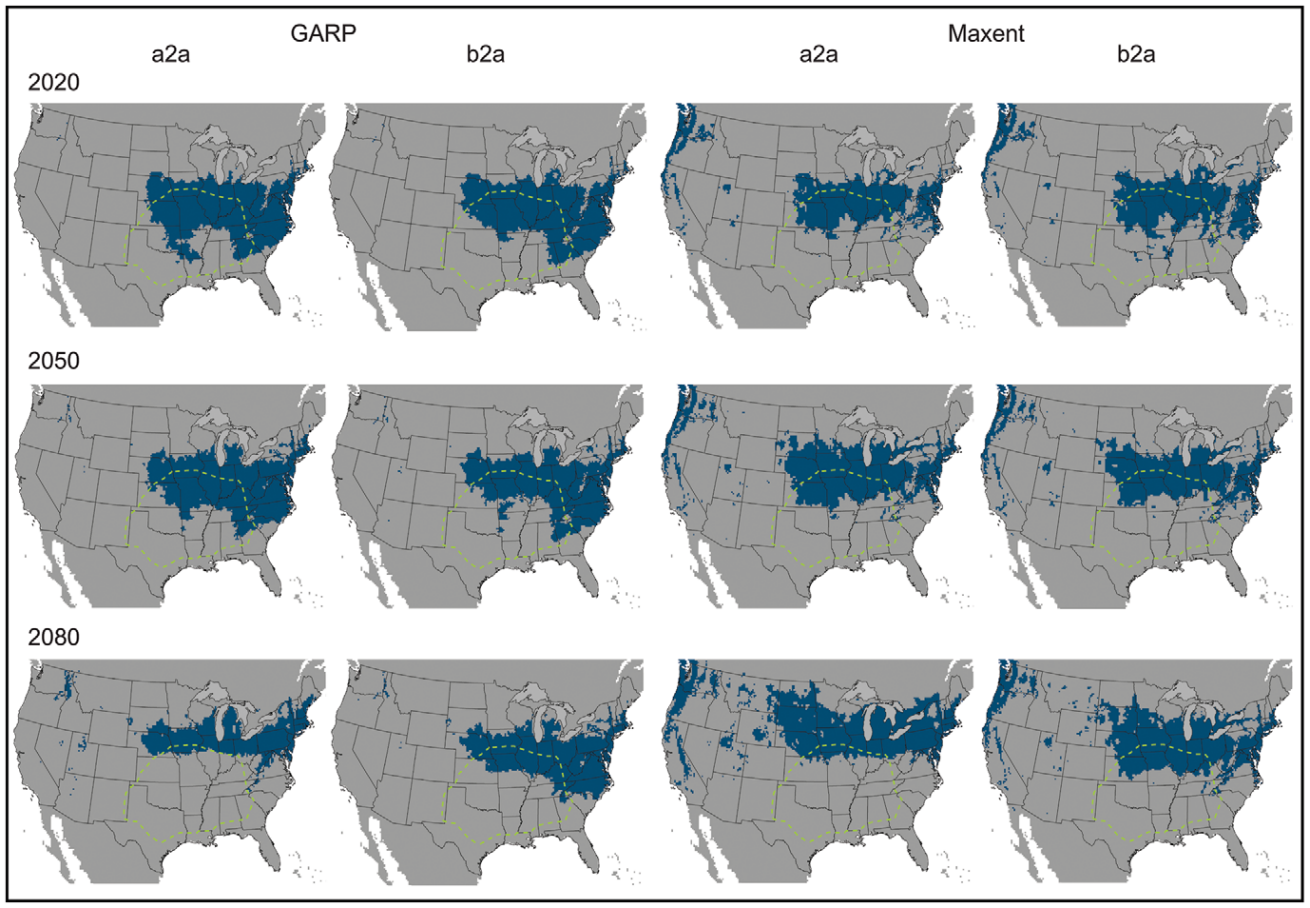
Future niche modeling results for three time slices: 2020, 2050, and 2080. GARP models are depicted on the left, with Maxent models on the right. Two climate change scenarios were utilized: a2a (liberal) and b2a (conservative). The lime green dotted polygon indicates the distribution of *L. reclusa* according to arachnologists, as was depicted in Fig. 2B. Suitable habitat for the brown recluse shifts northward with time. A threshold has been applied, allowing for a maximum of five percent omission error based on presence data. Results are depicted in USA Contiguous Albers Equal Area Conic map projection.

**Figure 4:**
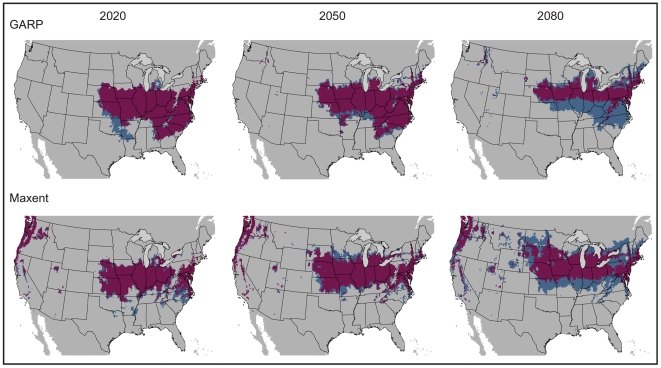
Model agreement between climate change scenarios per time slice. GARP results are depicted above, with Maxent models below. The two climate change scenarios (a2a and b2a) are compared, with area of overlap indicated in maroon, for the three time slices: 2020, 2050, and 2080. A threshold has been applied, allowing for a maximum of five percent omission error based on presence data. Results are depicted in USA Contiguous Albers Equal Area Conic map projection.

While the above analyses facilitate relatively straightforward bite diagnoses based on geography, this situation may become more ambiguous in the near future. Recent shifts in the geographic ranges of many species as a function of climate change have been observed on a global scale [17], [41]–[46]. The Earth's climate is predicted to warm at a rate of about 0.2°C/decade for the next two decades in many global climate models [47]. Organisms are expected to respond to these changes by habitat tracking, extinction, or, less likely, adaptation. Analysis of the effects of climate change on *L. reclusa* using both liberal (a2a) and conservative (b2a) forecasts of change suggests a northward shift and spreading to the east and west in the geographic space where the current niche conditions are met (Fig. 3). In the Northern Hemisphere, northward shifts in species' distributions as a result of environmental change have already been observed in other taxa, including birds, mammals, and butterflies [48]. Both the liberal (a2a) and conservative (b2a) future climate scenarios indicate new states could be invaded, including parts of Nebraska, Minnesota, Wisconsin, Michigan, South Dakota, Ohio, and Pennsylvania (Fig. 3). The two climate change scenarios (a2a and b2a) did not produce dramatically different model results (Table 1, Fig. 4); however, divergence between the scenarios increased with time (e.g., greatest divergence with the 2080 time slice) using both GARP and Maxent.

**Table 1:** Percent of niche overlap between the two climate change scenarios (a2a and b2a) for three time slices: 2020, 2050, and 2080.

Algorithm	2020	2050	2080
**Maxent**	81.42	78.76	56.75
**GARP**	85.57	83.90	46.45

**Figure 5:**
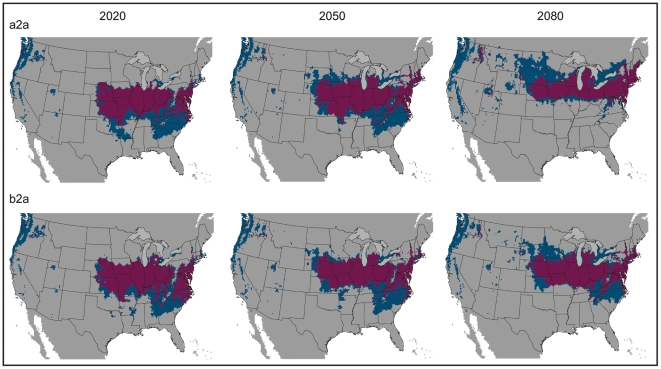
GARP-Maxent model agreement for each time slice and scenario. Maroon signals agreement; thus, blue areas are where Maxent predicted suitable habitat, but not GARP, or vice versa. A threshold has been applied, allowing for a maximum of five percent omission error based on presence data. Results are depicted in USA Contiguous Albers Equal Area Conic map projection.

The amount of suitable area did not differ by more than 7.12 percent between present and future projections, but the shape and position of the niche in geographic space did change (Table 2). Maxent and GARP predicted a similar percentage of suitable area within the United States, although available suitable area increased with time in Maxent models and decreased with time in GARP models (Table 2). This divergence likely reflects differences in the underlying mechanics of the two algorithms. Niche modeling algorithms commonly produce results that differ [49]–[51], which has generated support for an “ensemble” approach to predicting species distributions. Several strategies for handling model variability have been proposed in ensemble forecasting: (i) a single best model (providing the best fit to available data) is chosen, (ii) models are presented individually, and (iii) models are combined into a consensus prediction via model agreement, model central tendency (e.g., mean), or probability density functions [52]. Here, we present maps of model agreement between GARP and Maxent projections onto climates corresponding to three time slices: 2020, 2050, and 2080 (Fig. 5). In addition, we summarize separately, by algorithm and climate scenario, future predicted distributions to highlight differences between model outputs, since ensemble forecasting is not problem free, presenting issues such as masking model errors [53] (Fig. 3).

**Table 2:** Percent of suitable niche space for *L. reclusa*, based on the projected geographic region.

		Future					
		a2a			b2a		
Algorithm	Present	2020	2050	2080	2020	2050	2080
**Maxent**	13.68	12.28	16.14	19.26	13.55	14.31	16.80
**GARP**	15.89	13.57	13.49	8.77	12.78	12.28	11.40

**Figure 6:**
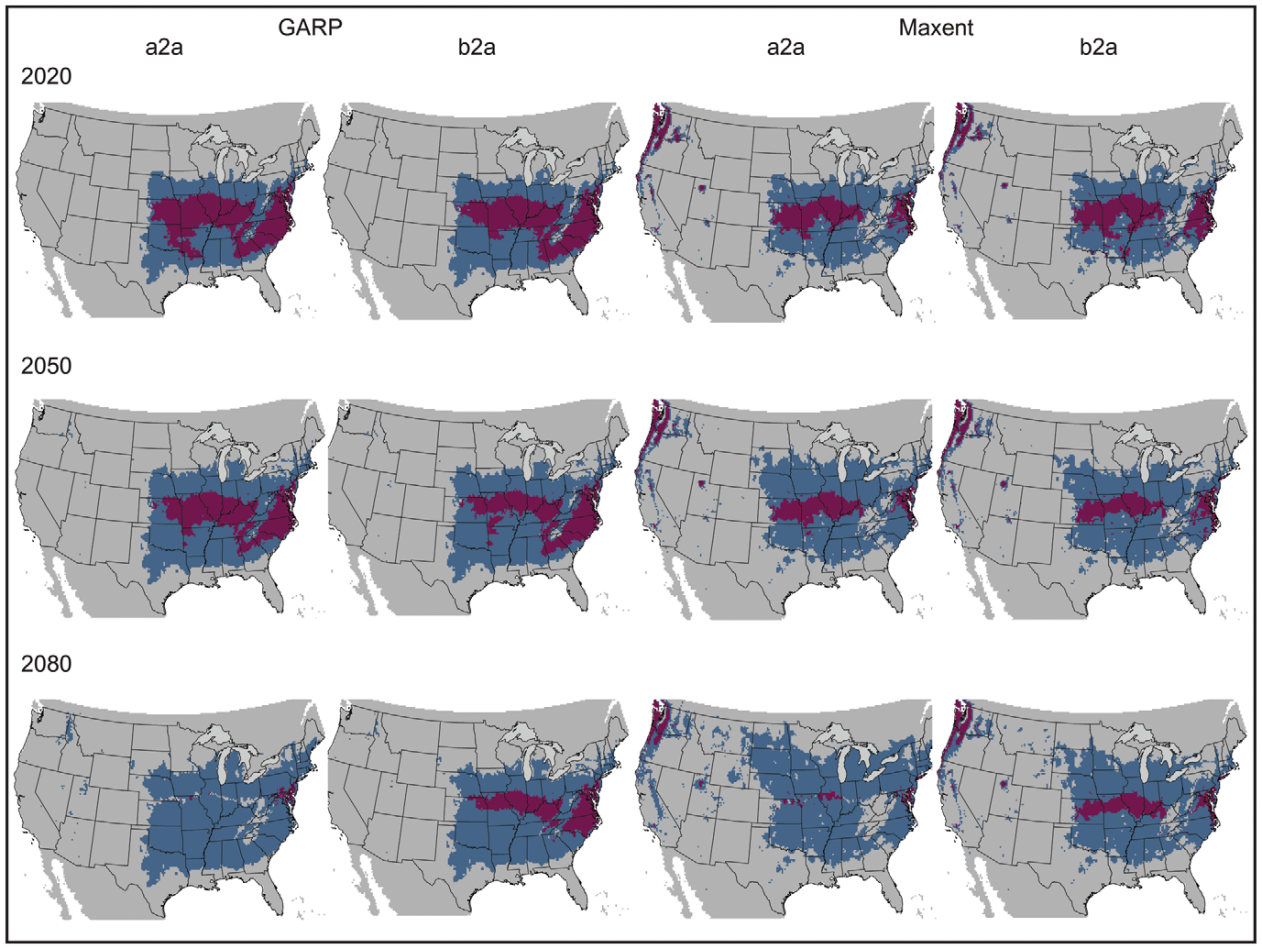
Niche overlap between extant and future models. GARP results are depicted on the left, with Maxent models on the right. Three time slices: 2020, 2050, and 2080 are illustrated for two climate change scenarios: a2a (liberal) and b2a (conservative). Area of overlap between the extant and future models is portrayed in maroon. A threshold has been applied, allowing for a maximum of five percent omission error based on presence data. Results are depicted in USA Contiguous Albers Equal Area Conic map projection.

As would be expected, the percent area of overlap between the present and future projected niches is negatively correlated with year (Table 3, Fig. 6). GARP models generally had a greater overlap percentage as compared to Maxent models, with an average overlap of 24.93% for GARP compared to 17.31% for Maxent in the a2a scenario, and 28.62% for GARP versus 22.38% for Maxent in the b2a scenario.

**Table 3:** Percent of niche overlap between present and future models.

	a2a			b2a		
Algorithm	2020	2050	2080	2020	2050	2080
**Maxent**	28.44	18.62	4.88	32.92	18.94	15.27
**GARP**	43.03	30.38	1.38	37.61	27.89	20.36

While there appears to be a dispersion corridor for *L. reclusa* (Fig. 6; area of overlap), there is also the potential for extinction (entirely or in portions of its range) if environmental change occurs too quickly (given niche conservatism [54]–[57]) and/or the species is not able to track its preferred habitat effectively. The future niche models assume unlimited dispersal ability, which may or may not be a valid assumption for this species. Dispersion of *L. reclusa* is limited by its inability to balloon (i.e. be carried aloft by air currents) [4], and thus the species may not track suitable habitat northward in step with changing climate. However, the partial synanthropy of the brown recluse, which can disperse with human movement, may override a low biological dispersion/dispersal potential.

The complexity of climatic processes leads to uncertainty regarding how the Earth's biota will respond to climate change. ENM serves as a powerful tool to study these potential adjustments [14], [28]–[32], [58]–[59]. The analyses presented herein illustrate abiotic constraints to the distribution of the brown recluse and highlight the potential for this species to move beyond the region it currently inhabits as climate changes; these data are of relevance to health professionals and the public, both at present and in the future.

We show that the geographic region representing suitable habitat for the brown recluse may be considerably different in the future from that of today. By 2080 perhaps only 5 percent of the area characterized as suitable today will still fall under this category. Newly suitable areas may encompass portions of Wisconsin, Michigan, Indiana, Ohio, Pennsylvania, New York, Nebraska and South Dakota. These states do not currently deal with this species, at least on a large scale, which may create a public health concern.

If a similar degree of niche displacement occurs across the myriad species that exist globally, the biodiversity of this planet will be significantly impacted. However, we are just beginning to understand the mechanics and consequences of these predicted changes; we understand to an even lesser degree the consequences on human health due to changes in the distributions of disease vectors and pathogens. As in the present study, we can overcome some of this uncertainty by using ENM to identify future potential high-risk areas for disease vectors and hosts, explore parasite-reservoir associations, and aid in planning vector-control strategies. ENM is useful for studying the complex dynamics of environment and biota over time and estimating distributional changes to medically important species [this paper], [ 28]–[32], pests [11], [60]–[62], and those organisms in need of conservation [58]–[59], [63]–[64].

## Materials and Methods

### (a) Ecological niche modeling

Predictions about a species' geographic distribution are built using the correspondence between information about the presence of a species and the associated environmental characteristics from its known range, analyzed via computer algorithms [33]–[34], [65]. The methodology is most often described as ecological niche modeling (ENM), habitat modeling, or species distribution modeling [66]–[68]. These are not identical, and disagreements regarding the conceptual background and ecological interpretation of resulting models exist [13], [69]–[70]. Here we employ ENM and two of the most common applications of projecting the resulting niche model on different spatial (geography) and temporal (future climates) domains.

In ENM, species geographic occurrence points and predictor variables (usually climatic) are used in correlative approaches to make inferences about the ecological requirements for a species. These requirements are often referred to as the niche of a species, defined as the set of tolerances and limits in multidimensional space that constrain where a species is potentially able to maintain populations [71]. The modeled niche can be projected onto extant and future climatic landscapes by identifying the current set of favorable conditions and selecting for those same climatic parameters on future maps. This technique is successful because, at broad scales, abiotic factors are generally sufficient to characterize the distribution of a species [54], [72]–[73]. Furthermore, the niche of a species is usually conserved [54]–[57], suggesting adaptation to new niche space is unlikely, particularly under the short time scales analyzed.

A criticism levied on projecting upon changed landscapes is that modified interactions between species may influence potential distribution more so than abiotic factors [74]–[75]. However, as mentioned, abiotic variables appear to successfully predict paths of species invasions and geographic distributions at broad scales [54], [72]–[73]. The future models can be thought of as representing null expectations, sans interspecific biotic parameters and assuming unlimited dispersal ability. These null models are useful for exploring “what if” questions [15], [18].

### (b) Modeling algorithms

GARP and Maxent were chosen for niche model building, as both programs are designed for predicting species' distributions when only presence data are available [76]–[77].

The Genetic Algorithm for Rule-set Prediction (GARP) is a machine-learning algorithm that works in an artificial-intelligence framework [33]. Rules are created through simple “IF <condition1> <condition2> … THEN <prediction>” statements. Once a rule is selected, it is applied to the training data (half the points input into the program) and allowed to evolve to maximize predictive accuracy. The change in predicted accuracy between iterations is used to evaluate whether a rule should be included within the model. We used a desktop version of GARP (Desktop GARP 1.1.3) [78], activating the internal testing feature (i.e., 50% of the input data were used to evaluate model quality within GARP). We ran 100 models with a 0.01 convergence limit and max iterations of 1000. Due to the random-walk nature of GARP, we implemented the “best subsets” procedure [35] to retain 10 models based on two error statistics, omission (excluding known presence data) and commission (including areas without confirmed presence of species, but which are potentially habitable). A soft omission threshold was used so that 20% of models with the lowest omission error were retained; those models with intermediate levels of commission were then chosen from this subset. The 10 best models were summed in ArcMap 9.3 (ESRI, Redlands, CA) to create a model agreement map in GIS grid format.

Maxent is also a machine-learning method that estimates a probability distribution for species' occurrences by finding the distribution of maximum entropy (that which is closest to uniform), subject to constraints defined by the environmental parameters input into the model [34]. The default features of Maxent v. 3.1.1 were utilized, including random test percentage = 0, regularization multiplier = 1, and maximum number of background points = 10000. We also took advantage of the “remove duplicate presences” function. The linear, quadratic, product, threshold, and hinge feature types were enabled. We converted the floating-point output models of Maxent into integer grids (retaining first 3 decimals), which are easier to manipulate in a GIS-framework, using the Raster Calculator in ArcMap 9.3.

### (c) Distributional data

Brown recluse occurrence data were obtained from the American Museum of Natural History, the Museum of Comparative Zoology, and from the Nationwide Brown Recluse Challenge and a survey of Georgia by one of us (RSV). Alex Maywright and Zuleyma Tang-Martinez (University of Missouri, St. Louis) kindly provided a dataset from Illinois (from the Illinois Natural History Survey), and Gail Stratton (University of Mississippi) generously donated a dataset from a survey in northern Mississippi.

Locality information was georeferenced using the point-radius method [79], where each locality was treated as a circle with a point in the center. The radius represents the maximum distance from the point within which the locality is expected to occur. All occurrence points were georeferenced, excluding the Mississippi dataset that had been assigned coordinates with a GPS. Error was allocated to the GPS data points using the MaNIS/HerpNet/ORNIS Georeferencing Calculator [79]. Georeferencing was primarily conducted using BioGeomancer [80], since most localities simply referred to a town and state. The center of the town was manually determined using the underlying topographic map function, and the error was adjusted within BioGeomancer to include the full extent of the town. When presented with a specific street address, the exact address was georeferenced, and the extent of the street was used to calculate error. Localities described with offset distances (e.g., “1 mi south of Rolla, Missouri”) were georeferenced by measuring the extent and center of the named place (usually a town) in Google Earth 5.0. These measurements were then imported into the MaNIS/HerpNet/ORNIS Georeferencing Calculator to find the geographic coordinates and error associated with them. Any obviously inaccurate and/or dubious locality information was not georeferenced.

Only those locality points with less than 18 km spatial uncertainty were retained, totaling 240 spatially unique records. Model training occurred with 126 records; the other portion was set aside for model validation. The error in the locality data should not significantly influence model performance [81]. Note that verifiable sink populations (i.e., spot records) were not selected for model building for fear of biasing results; thus, the Atlantic seaboard records mentioned from the Nationwide Brown Recluse Challenge [40] were not employed in modeling.

### (d) Predictor data

Niche models for the present-day distribution were constructed using seven climatic variables from WorldClim v. 1.4 [82], including annual mean temperature, mean diurnal range, maximum temperature of warmest month, minimum temperature of coldest month, annual precipitation, precipitation of wettest month, and precipitation of driest month. These seven variables capture the climatic dimensions most likely to limit the distributional extent of the species, and they have been used in other studies to positive ends [e.g.], [ 26,83]. The data were downloaded in the form of 10 arc-minute bioclimatic GIS grids, mirroring the resolution of our occurrence data. The layers were clipped to the training region of the study, which included the area between the Rocky and Appalachian Mountains, USA (i.e. the Midwest). This region was chosen because it (i) encompasses the entire range of the brown recluse as determined from previous studies, and (ii) represents an area most likely accessible to the species (i.e., within its “M” domain but including area thought to be unsuitable, *sensu* [84]).

The same seven predictors were employed in forward modeling. Future climate data were downloaded in the form of 10 arc-minute grids from WorldClim v. 1.4 [82]; layers were calibrated and statistically downscaled using the WorldClim data for current conditions. The future environmental parameters were derived from the Canadian Centre for Climate Modeling and Analysis (CCCma) Second Generation Coupled Global Climate Model (CGCM2) under the IPCC3 A2 and B2 emission scenarios [85]. We used a liberal (a2a) and conservative (b2a) scenario of socio-economical and associated green house gas changes for three time slices: 2020, 2050, and 2080. The A2 and B2 storylines assume heterogeneous world development, as opposed to globalization. Driving forces in the a2a storyline are high population growth rates, increased energy and land-use changes, and slow technological change. Conversely, the B2 storyline simulates slower population growth rates and land-use changes and more technological innovations.

The data were imported into DIVA GIS 7.1.6 to correctly convert native BIL layer formats to ESRI grid files. Arc Macro Language (AML) code (available at www.worldclim.org) was then run to generate the same set of bioclimatic variables used for current modeling. The ability to match the bioclimatic variables used for present-day modeling is why we chose the CCma climate model.

### (e) Model analysis and validation

Model quality was assessed with (i) an omission error test, which examines false negatives or the number of test occurrences predicted absent by the niche models [35], (ii) by comparison to expert opinion [4], and (iii) with the partial Receiver Operating Characteristic analysis (partial ROC) [35]–[36]. The area under the curve (AUC) in ROC analyses is a threshold-independent measure of model performance as compared to null expectations. By implementing a threshold on the 1-omission error (y) axis, calculation of partial ROC is restricted to the region of high model sensitivity (low omission error). To compare model ROC AUC ratios with null expectations, the dataset was bootstrapped and a Z value (standard normal approximation) obtained. We used a Visual Basic routine developed by N. Barve (University of Kansas) to calculate AUC ratios, performing 1000 iterations with the omission threshold set at five percent [36].

To facilitate comparison between predictions, we reclassified the model agreement (GARP) and continuous (Maxent) outputs to simple 0 and 1 values (0 = unsuitable habitat, 1 = suitable habitat). All models were reclassified to presence/absence pixels within ArcMap 9.3 using threshold values that allowed a maximum of five percent omission error based on the presence data available. Calculation of area predicted present was performed using the Zonal Statistics function of ArcMap 9.3 and the USA Contiguous Albers Equal Area Conic map projection. The ArcMap Raster Calculator was used to determine area of overlap between niche models.
